# Acute rifampicin-associated interstitial tubulopathy in a patient with pulmonary tuberculosis: a case report

**DOI:** 10.1186/1752-1947-7-106

**Published:** 2013-04-17

**Authors:** Silvia Rosati, Chiara Cherubini, Fabio Iacomi, Konstantinos Giannakakis, Laura Vincenzi, Giuseppe Ippolito, Fabrizio Palmieri

**Affiliations:** 1 Clinical Department, “L. Spallanzani” National Institute for Infectious Diseases I.R.C.C.S., Rome, Italy; 2 Nephrology and Dialysis Unit for Infectious Diseases, S. Camillo Hospital, Rome, Italy; 3 Department of Experimental Medicine, Sapienza University, Rome, Italy; 4 Epidemiological Department, “L. Spallanzani” National Institute for Infectious Diseases I.R.C.C.S., Rome, Italy

**Keywords:** Acute renal failure, Kidney biopsy, Rifampicin, Tubulointerstitial nephritis

## Abstract

**Introduction:**

Rifampicin is one of the most effective antibiotics for treating tuberculosis, but it has been associated with adverse reactions, such as nephrotoxicity, sometimes resulting in acute renal failure with oligoanuria, and hepatotoxicity. Although deterioration of renal function, determined by acute tubulointerstitial nephritis and/or acute tubular necrosis, typically appears in patients receiving intermittent rifampicin therapy, some authors have also reported cases occurring during continuous rifampicin therapy.

**Case presentation:**

We describe the case of acute renal failure with polyuria occurring in a previously healthy 50-year-old Caucasian man undergoing continuous therapy with rifampicin for culture-confirmed pulmonary tuberculosis. The patient was admitted to the L. Spallanzani National Institute for Infectious Diseases, Rome, Italy, with a 1-month history of coughing, fever and weight loss. After 6 weeks of standard antituberculous treatment, progressive deterioration of his renal function was observed: creatinine levels rose from 38.9μmol/L to 318.2μmol/L and urine volume also progressively increased to reach a state of true polyuria (8 to 10L of urine per day). He was diagnosed with suspected acute rifampicin-induced renal failure. A renal biopsy showed focal segmental glomerulosclerosis associated with acute tubulointerstitial nephritis. Rifampicin was discontinued with excellent results: after 15 days his renal function began to improve and his serum creatinine values returned to normal.

**Conclusion:**

A high index of suspicion for rifampicin-associated acute renal failure should be maintained in patients with pulmonary tuberculosis who develop progressive deterioration of renal function during treatment with rifampicin. Early diagnosis and discontinuation of rifampicin are of fundamental importance for recovering renal function.

## Introduction

Rifampicin is one of the most effective antibiotics used for treating tuberculosis, but it has been associated with adverse reactions such as nephrotoxicity [1-4], sometimes resulting in acute renal failure (ARF), and hepatotoxicity [1,2]. Although deterioration of renal function, determined by acute tubulointerstitial nephritis and/or acute tubular necrosis, typically appears in patients receiving intermittent rifampicin therapy, some authors have also reported cases occurring during continuous rifampicin therapy [1-4]. Retrospective studies have been made to assess the prevalence, and the clinical and biochemical features, of ARF following rifampicin treatment [4-6]. ARF related to administration of rifampicin is generally associated with oligoanuria, whereas our patient showed polyuria.

We describe a case of ARF with polyuria occurring in a patient receiving continuous administration of rifampicin for pulmonary tuberculosis; biopsy findings revealed focal segmental glomerulosclerosis associated with acute tubulointerstitial nephritis.

## Case presentation

**Figure 1:**
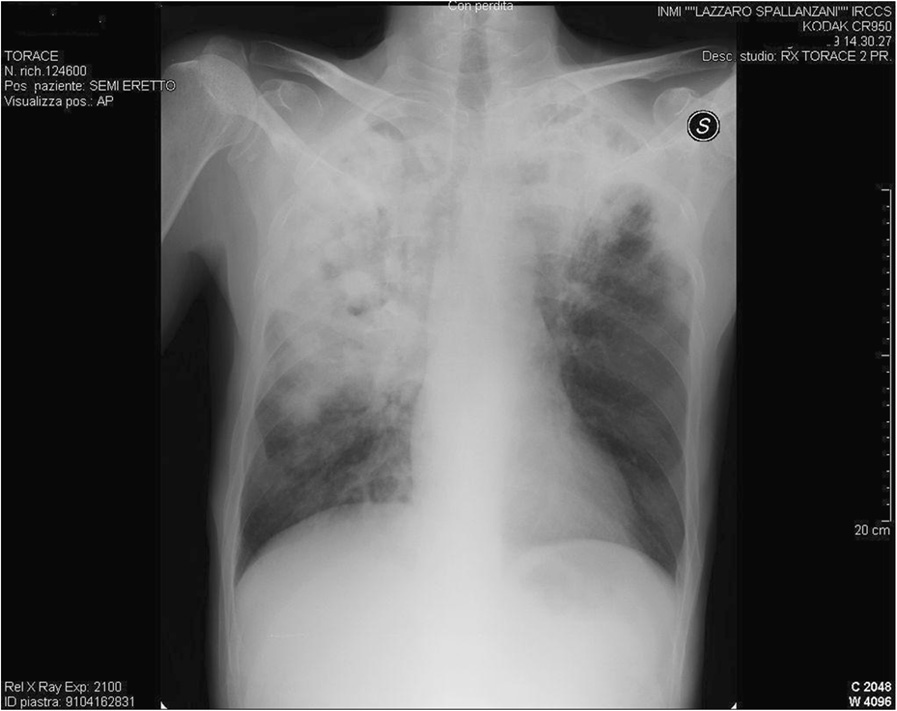
Posteroanterior chest radiography: extensive upper right subclavian and apex left subclavian infiltrates with cavitation.

**Figure 2:**
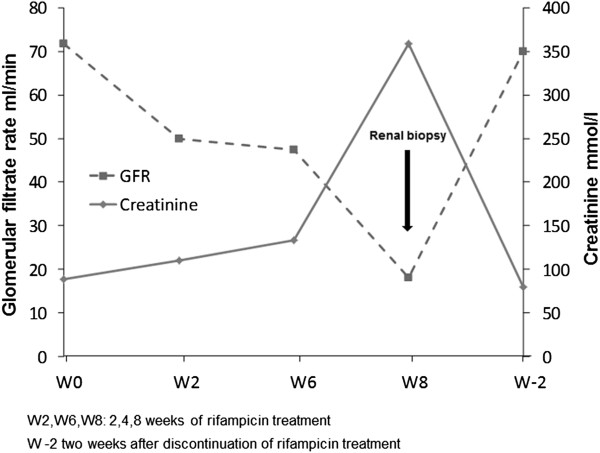
**Time relationship between rifampicin treatment, serum creatinine level and glomerular filtration rate.** W2/W6/W8: 2/6/8 weeks of rifampicin treatment; W-2: 2 weeks after discontinuing rifampicin treatment. GFR; Glomerular filtration rate.

**Figure 3:**
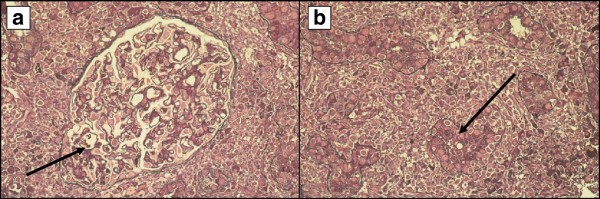
**Histology of renal biopsy (periodic Schiff-methenamine staining on a semi-thin section).** (**a**) Glomerulus with a moderate, initial segmental sclerotic (arrow) lesion (400× magnification). (**b**) Intense interstitial inflammation with lymphocytes and granulocytes (arrow) showing invasion and focal destruction of the tubular epithelium (200× magnification).

A previously healthy 50-year-old Caucasian man was admitted to the L. Spallanzani National Institute for Infectious Diseases in Rome, Italy, with a 1-month history of coughing, fever and weight loss. Chest radiography demonstrated “extensive upper right subclavian and apex left subclavian infiltrates with cavitation” (Figure 1). Sputum samples showed numerous acid-fast bacilli and ribonucleic acid amplification-based assay from induced sputum was positive for *Mycobacterium tuberculosis* and the culture later grew susceptible *M. tuberculosis*. The purified protein derivative skin test was reactive (10mm). The patient tested negative for human immunodeficiency virus. He began therapy with daily rifampicin (600mg), isoniazid (300mg), pyrazinamide (1500mg) and ethambutol (1200mg). Results of initial laboratory testing including complete blood count, electrolytes and liver and kidney function tests were within normal ranges. On day 42 after admission (still sputum-smear positive), progressive deterioration of renal function was observed. Creatinine levels progressively rose from 38.9μmol/L on admission to 318.2μmol/L (normal, 44.2 to 123.7μmol/L), with a creatinine clearance of 18mL/minute (Figure 2) and an increase of urinary sodium (Na+ 285mEq/24hours). Urine volume also progressively increased to reach a state of a true polyuria (8 to10 L of urine per day). Urine examination results were significant, showing 300mg/L of protein with a total excretion of 2500 to 4000mg of protein/day, 200mg/dL of glucose with a total excretion of 1.6 to 2.1g of glucose/day, 50 to 60 white blood cells/high power field and sterile leukocyturia (with sterile urine culture), considered a marker of interstitial nephritis. No eosinophils were present in the urine: specific weight was 1005 to 1007. A blood test indicated a hemoglobin level of 8.70g/dL (normal, 12 to 18g/dL), platelet count of 522 × 10^3^/μL (normal, 80 to 400 10^3^/μL), blood urea nitrogen of 0.29mmol/L (normal, 0.04 to 0.18mmol/L) and serum creatinine of 318.2μmol/L. He began modified antituberculous treatment consisting of isoniazid 300mg once daily, pyrazinamide 1500mg three times weekly, ethambutol 1000mg three times weekly, rifampicin 600mg once daily and moxifloxacin (400mg once daily) but his renal function did not improve. Venous blood gas analysis showed metabolic acidosis (pH 7.30, bicarbonate 19mmol/L, base excess −5) and hypokalemia (K+ 2.8mEq/L). Immunoglobulin testing of the serum showed an increase of immunoglobulin As and immunoglobulin Gs; anti-double stranded deoxyribonucleic acid (DNA) antibodies were positive (1:160), antinuclear antibodies were negative and complement factors C3 and C4 levels were normal. Abdominal ultrasonography showed “…kidney increased in volume, with disappearance of the normal cortico-medullary differentiation…”. The patient was treated with 7 to 8L/day of liquids via intravenous infusion; acidosis and hypokalemia were corrected and erythropoietin was administered for anemia. On day 55, his serum creatinine was 358.9μmol/L, blood urea nitrogen was 0.25mmol/L and polyuria persisted. Serum test results for the antidiuretic hormone were within the normal range. Based on previous reports of similar cases, a diagnosis of suspected acute rifampicin-induced renal failure was made. A renal biopsy showed focal segmental glomerulosclerosis associated with acute tubulointerstitial nephritis (Figure 3a-3b). Rifampicin was discontinued and the response was excellent: no further therapy was required (steroids or hemodialysis). Renal function began to improve and 15 days after discontinuing rifampicin, serum creatinine values returned to normal (Figure 2). The polyuria gradually regressed and after 20 days, diuresis and venous blood gas analysis returned to normal.

## Discussion

There is currently a greater prevalence of acute post-rifampicin renal failure due to the high spread of tuberculosis. ARF usually occurs in patients who receive intermittent regimens (two or three times weekly), but some authors have reported [1-4] ARF after daily treatments. Retrospective studies have been made to assess the prevalence, and the clinical and biochemical features, of ARF following rifampicin treatment [4-6]. ARF related to administration of rifampicin is generally associated with oligoanuria, whereas our patient showed polyuria. Various mechanisms of rifampicin-associated ARF have been postulated [6] and it is difficult to determine the incidence of ARF among all patients treated with rifampicin. The mechanism of renal damage is thought to be due to allergic reactions to rifampicin or one of its metabolites causing allergic interstitial nephritis. Immunogenicity of rifampicin has been demonstrated by the presence of rifampicin-dependent antibodies in serum, especially immunoglobulin M [6-9], but the relationship between anti-rifampicin antibodies and the development of renal failure is not clear; circulating rifampicin-dependent antibodies have been observed in patients on treatment with rifampicin without any evidence of renal disease [8]. Conversely, not all patients on treatment with rifampicin who develop renal failure have demonstrable circulating antibodies [10]. Our patient showed focal segmental glomerulosclerosis associated with severe acute tubulointerstitial nephritis with polyuria which regressed upon discontinuation of the drug. There was no need for therapy with corticosteroids or hemodialysis treatment. No cases of polyuria are reported in the data supplied by the literature or retrospective studies.

## Conclusion

Acute interstitial nephritis is becoming an important cause of acute, but reversible, renal failure and drug hypersensitivity is the most common and important etiological factor for this condition. The pathogenesis of tubular necrosis has not yet been defined. Renal biopsy is the “gold standard” for the diagnosis. A high index of suspicion for rifampicin-associated ARF should be maintained in patients with pulmonary tuberculosis who develop progressive deterioration of renal function during treatment with rifampicin. Early diagnosis and discontinuation of rifampicin are of fundamental importance for recovering renal function. We believe that the description of this case may contribute to clinical experience due to the peculiarity of the relation between the development of polyuria and administration of rifampicin.

## Consent

Written informed consent was obtained from the patient for publication of this case report and any accompanying images. A copy of the written consent is available for review by the Editor-in-Chief of this journal.

## Competing interest

The authors declare that they have no competing interests.

## Authors’ contributions

SR, FI, LV, and FP diagnosed and followed the patient. SR acquired clinical data. CC and KG performed and interpreted the histological examination. SR prepared the manuscript and FP and GI evaluated the draft and suggested revisions. All authors read and approved the final manuscript.
